# Long-term outcome of basilar stenosis in Erdheim–Chester disease: A case report

**DOI:** 10.1097/MD.0000000000004813

**Published:** 2016-09-09

**Authors:** Stéphane Mathis, Gaëlle Godenèche, Julien Haroche, Serge Milin, Adrien Julian, Aline Berthomet, Clément Baron, Paola Palazzo, Jean-Philippe Neau

**Affiliations:** a Department of Neurology, CHU of Poitiers, University of Poitiers, Poitiers; b Department of Internal Medicine and French reference Center for Rare Auto-immune and Systemic Diseases, Assistance Publique–Hôpitaux de Paris (AP-HP), Pitié-Salpêtrière Hospital; c Université Pierre et Marie Curie, UPMC University Paris 6, Paris; d Department of Pathology, CHU of Poitiers, University of Poitiers, Poitiers, France.

**Keywords:** basilar, Erdheim–Chester disease, histiocytosis, infiltration, stroke

## Abstract

**Background:**

Erdheim–Chester disease (ECD) is a rare form of non-Langerhans cell histiocytosis. This inflammatory myeloid neoplasm is frequently complicated by neurological symptoms, but stroke is an exceptional manifestation of this disease.

**Methods:**

We report the case of a 59-year-old woman who presented a vertebrobasilar stroke secondary to infiltration and severe stenosis of the basilar artery, improved after interferon-alpha therapy. We performed a review of the relevant literature and reported the few other cases described.

**Results:**

With our patient, we have found only 7 observations of cerebrovascular disorder in ECD. Most of them had supravascular arteries involvement.

**Conclusion:**

Stroke is a rare treatable and potentially reversible complication of ECD. The pathophysiological processes explaining stroke in this disease are uncertain, but periarterial stenosis of cerebral arteries may be a mechanism.

## 1 Introduction

Histiocytosis is defined as a nonmalignant disorder due to abnormal accumulation and behavior of cells of the mononuclear phagocytic system (or “histiocytes”). Histiocytes were first described in the skin by Paul Langerhans (Langerhans cells) who initially thought that these dendritic processes were part of the nervous system.^[1]^ However, as other histiocytes, these mononuclear phagocytes (arising from the myeloid hematopoietic lineage) are antigen-presenting cells ingesting foreign material, processing their protein antigens and presenting them to lymphocytes to initiate the immune response: they normally participate in wound and tissue repair, and have key roles in initiation as well as in the regulation of inflammation.^[2]^ Histiocytic disorders are usually defined by their constitutive cell types, with 4 subtypes (“Langerhans cells histiocytosis,” “non-Langerhans cells histocytosis,” “hemophagocytic lymphohistiocytoses,” and “histiocyte lineage-related malignancies”).^[3]^ Some authors have proposed to classify these disorders as “inflammatory myeloid neoplasms.”^[4]^

Erdheim–Chester disease (ECD), first described by Jakob Erdheim and William Chester in 1930 as “lipoid granulomatosis,”^[5]^ is a rare (up to 600 cases reported in the medical literature) non-Langerhans form of histiocytosis.^[6]^ Diagnostic criteria for ECD are based on characteristic pathological findings (foamy histiocytes infiltration of polymorphic granuloma and fibrosis and xanthogranulomatosis, with CD68^+^CD1a^−^ immunostaining), but also skeletal abnormalities (“bilateral and symmetric cortical osteosclerosis of the diaphyseal and metaphyseal parts of the long bones on X-ray” and/or “symmetric and abnormally intense labeling of the distal ends of the long bones of the legs, and in some cases of the arms, as revealed by ^99m^Technetium bone scintigraphy”), whereas this last criteria is not strictly required for the diagnosis.^[7]^ If bone infiltration by foamy histiocytes is important for the diagnosis of ECD, numerous other tissues may be involved such as skin, kidneys, lungs, pleura, liver, peritoneum, spleen, and small bowel, but also the central nervous system (CNS).^[8]^ CNS may be a target of histiocytes,^[9]^ but among all neurological manifestations of ECD, stroke has been exceptionally described.^[10–14]^ Herein we report the observation of a patient who developed stroke which contributed in making the diagnosis of ECD. We have also performed a review of similar observations reported in medical literature.

## 2 Case report

A 59-year-old woman was admitted to the department of Neurology for a sudden confusion with loss of vision. Her past medical history comprised of a total hysterectomy twenty-five years earlier (fibroma). She also complained of headaches and chronic asthenia for 5 years: at that time, medical investigations only found a chronic seromucous maxillary sinusitis treated by antibiotherapy, then endoscopic sinus surgery (the pathological study objective a nonspecific inflammation); however, she still complained of chronic headaches despite this treatment. At the age of 55, she had also developed refractory arterial hypertension and renal failure leading to the diagnosis of right renal artery stenosis and left renal artery occlusion: she was treated by surgery (stenting of the left renal artery, then right aortorenal bypass).

On clinical examination, there was cortical blindness, anosognosia for blindness, amnesia, and topographical disorientation suggestive of a Dide-Botcazo syndrome. There was no pyramidal sign nor cerebellar ataxia or cranial nerves involvement. No other clinical abnormality was reported. She still complained of chronic headaches.

**Figure 1:**
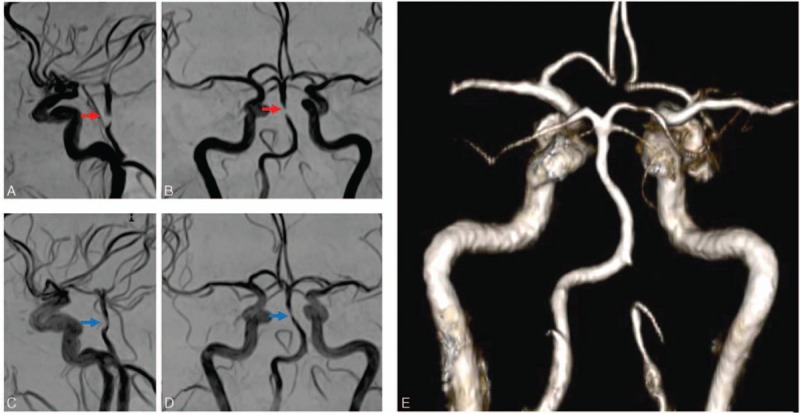
(A) and (B) (before interferon-α treatment): (A) contrast-enhanced MR angiogram; (B) DSA demonstrates basilar artery severe stenosis (red arrows). (C and D) (3 months after interferon-α treatment): (C) contrast-enhanced MR angiogram; (D) DSA demonstrates partial regression of the basilar artery stenosis (blue arrows). (E) (Two years after the initiation of interferon-α treatment): MR angiogram reconstruction shows the total recanalization of the basilar artery. DSA = digital subtraction angiography, MR = magnetic resonance.

**Figure 2:**
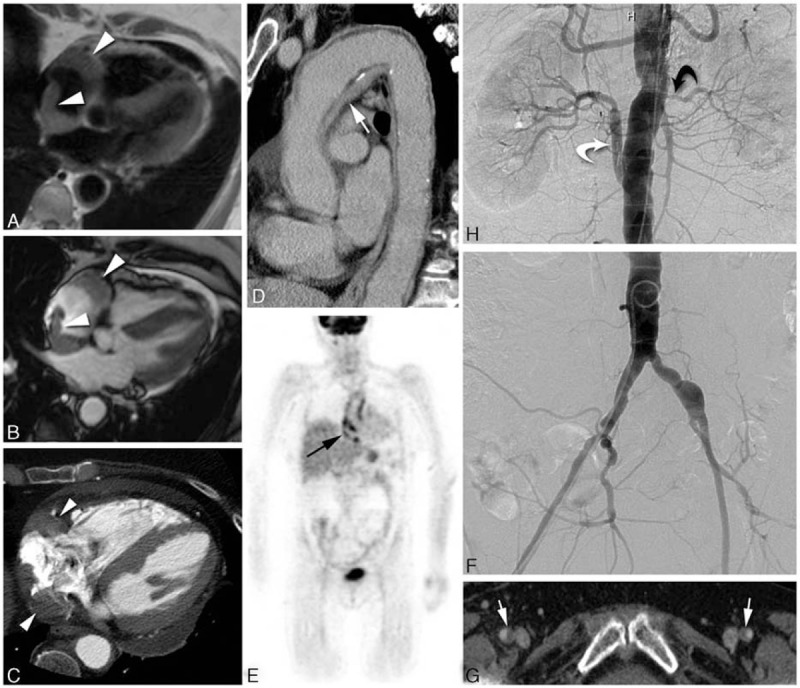
Diffuse cardiovascular involvement was observed. Cardiac MR (A, B) and contrast-enhanced cardiac CT **(**C**)** shows right atrial pseudotumor (white arrows). Contrast-enhanced thoracic CT **(**D**)** shows diffuse periaortic infiltration. ^18^FDG PET **(**E) shows abnormal periatrial and periaortic FDG uptake. DSA images **(**F) show diffuse aorto-iliac irregularity, left renal artery stent (black curved arrow), and right aorto-renal bypass (white curved arrow). Contrast-enhanced pelvic CT **(**G**)** shows bilateral femoral arteries infiltration. CT = computerized tomography, DSA = digital subtraction angiography, FDG = fluorodeoxyglucose, MR = magnetic resonance, PET = positron emission tomography.

Bilateral cerebral posterior infarction and hypodensity in the territory of the right postero-inferior cerebellar artery have been observed on brain magnetic resonance imaging (MRI) (and magnetic resonance angiography), with a severe narrowing of the basilar artery (closed to 90%) and a mild stenosis of the M1 and M2 segments of the left middle cerebral artery (Fig. 1A and 1B). Transcranial Doppler confirmed a severe stenosis of the basilar artery. Electrocardiogram and 24 hours electrocardiography were normal. Echocardiography (transthoracic and transesophageal) demonstrated a hypertrophic cardiopathy (with severe left ventricular hypertrophy and circumferential dilatation of the left atrium). No valvulopathy was observed, but there was a heterogeneous thickening of the interatrial septum (1.5 cm) and a hyperechogeneous thickening next to the right atrium (1.9 × 1.3 cm). Cardiac computed tomography angiography (and cardiac MRI) confirmed a right periatrial infiltration, and also found thickening of the wall of the whole aortic arch (with tardive enhancement of media) and pericardial effusion (Fig. 2). Whole-body ^18^fluorodeoxyglucose positron emission tomography (^18^FDG PET) found contrast-enhancement on the right and left atria, whole aorta, iliac arteries, and femoral arteries (Fig. 2E). Ophthalmological examination was normal, except a grade I hypertensive retinopathy.

Ancillary tests found hyperleukocytosis (13,000/mm^3^, with 8890 neutrophils/mm^3^), biological inflammation (C-reactive protein was 24 mg/L), with no monoclonal gammopathy. Viral and bacterial serologies, immunological (antinuclear, anticardiolipin, and anti-β_2_-glycoprotein 1 antibodies) and metabolic (cholesterol, triglyceride, glycosylated hemoglobin, homocysteine, thyroid stimulating hormone, and transaminases) tests were unremarkable. Cerebrospinal fluid analysis was normal (1 white cells/mm^3^; protein level at 46 mg/dL, for a normal value <45 mg/dL; normal glucose level). A bone marrow biopsy showed no abnormality.

**Figure 3:**
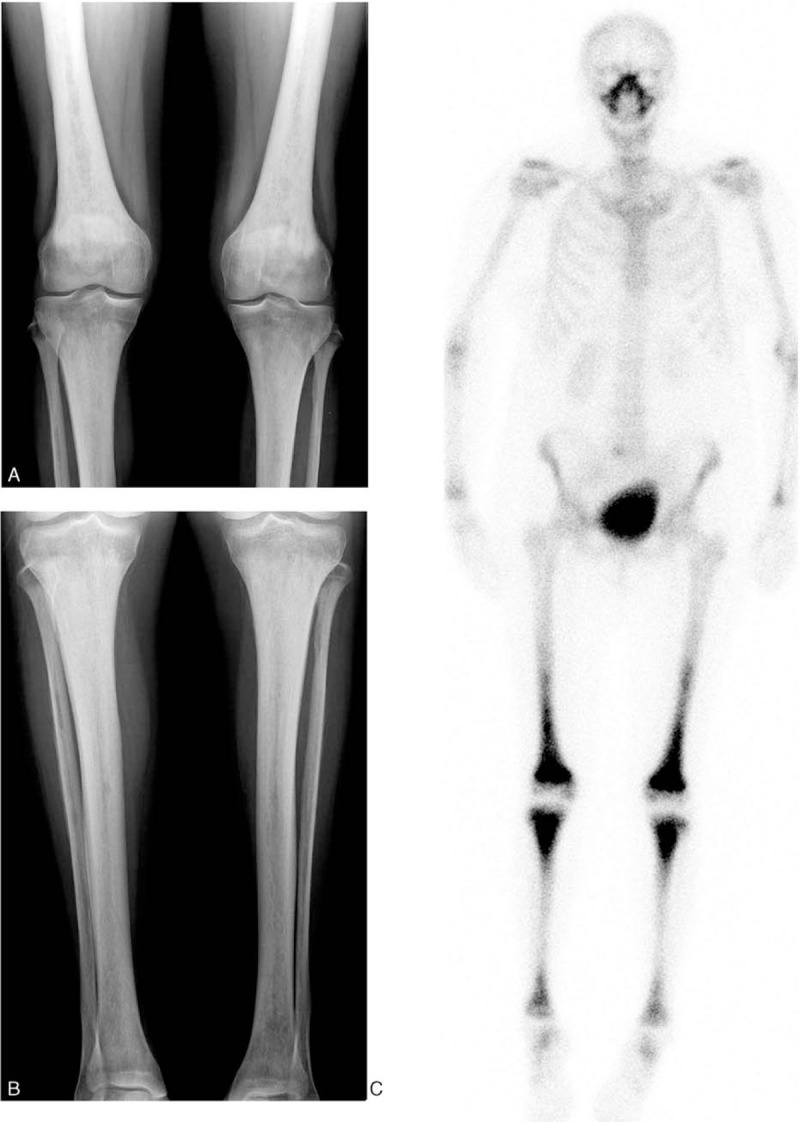
X-rays (A, B) shows bilateral and symmetric cortical osteosclerosis of the diaphyseal and metaphyseal regions in the long bones of the lower limbs with symmetrical hyperfixation on ^99m^Technetium bone scintigraphy (C).

Plain radiographs showed bilateral and symmetric cortical osteosclerosis of the diaphyseal and metaphyseal regions of the lower limbs; ^99^Technetium bone scintigraphy revealed a symmetric tracer uptake by the long bone of lower extremities (and also in maxillary and frontal bones) suggestive of the diagnosis of ECD (Fig. 3). After a second biopsy of the maxillary sinus, pathological study finally demonstrated a xanthogranulomatous infiltrate mainly composed by foamy histiocytes accompanied by fibrosis (stains for CD1a and S-100 were negative), confirming the diagnosis of ECD.

She was treated with interferon-α (3 million IU 3 times weekly, then a gradual increase of the dose up to 9 million IU 3 times weekly over the following months), with not only an improvement of the cognitive function (particularly visual function and memory) but also of headaches. A treatment by clopidogrel (75 mg/d) was proposed for stroke. After 3 months of treatment with interferon-α, we observed reduction of the stenosis of the basilar artery (50%) on MR-angiography (with a quite total recanalization 2 years after the onset of the treatment; Fig. 1). After 4 years, the neurological examination was still stable, but she developed insipidus diabetes (successfully treated with desmopressin).

## 3 Discussion

Most of patients with ECD are men (75%), mostly affecting during the fifth and sixth decades.^[7]^ Patients with ECD carry the V600E mutation of the *BRAF* gene in 54% of cases (as 57% of patients with Langerhans cell histiocytosis).^[15]^ ECD is not only associated with skeletal involvement in all patients, but is also a multisystemic disease with high mortality due to many extraskeletal manifestations: diabetes insipidus, xanthelasma, interstitial lung disease, bilateral adrenal enlargement, retroperitoneal fibrosis (with perirenal and/or ureteral obstruction), renal impairment, testis infiltration, cardiovascular disorders, and CNS involvement.^[7,8]^

In fact, CNS involvement is frequent in ECD, reported in 30% to 50% of cases.^[9,16,17]^ On 27 patients with ECD, diencephalic involvement was found in 23 patients, cerebellar involvement in 11 patients, and meningeal involvement in 9 patients.^[18]^ In 2006, in the largest review of medical literature (66 patients), the most frequent neurological manifestations were cerebellar (41%) and pyramidal (45%) signs, the other features being seizures, headaches, neuropsychiatric manifestations or cognitive impairment, sensory disturbances, and cranial nerve paralysis.^[9]^ In addition, systematic cranial MRI in 33 patients with ECD was normal in only 3 of them: most patients have 2 or more anatomical sites affected (hypothalamic-pituitary axis, brain, meninges, facial and skull bones, and orbits), even if they were asymptomatic.^[19]^ These neurological involvements lead to severe functional disability in almost all patients, and CNS involvement appears to be a major prognostic factor for ECD.^[7]^

Among these neurological manifestations, strokes are of rare occurrence, reported in only 1% of cases.^[16]^ With our patient, we have found only 8 observations of cerebrovascular disorder in ECD (Table 1).^[10–14,20,21]^ We have secondarily excluded the observation of Black et al^[20]^ because the patient also suffered from a familial hemiplegic migraine (probably the cause of the neurological deficit). Choi et al^[12]^ and Gauvrit et al^[10]^ reported transient ischemic attack; for the 5 other patients, the diagnosis of ECD was established after the diagnosis of stroke. However, three of these 5 observations are questionable. In the case of Amezyane et al^[11]^ a focal stroke was discovered on brain MRI, but was probably asymptomatic and did not explain the neurological deficit (due to a brain pseudonodular lesion of ECD); in the case of Mergancova et al,^[14]^ there were incomplete criteria of ECD; finally, Mélé et al^[21]^ described a patient with mixed histiocytosis (with both Langerhans and non-Langerhans forms in the same patient). Interestingly, in the other cases (as in the case of Mergancova et al^[14]^), vascular involvement (periarterial infiltration and sometimes stenosis) was observed in the supra-aortic arteries, whereas patients had no cardiovascular risk factors. However, in the radiological series of Drier et al,^[19]^ if 3 patients (on a total of 30) had such lesions, a stroke was observed in only one (corresponding to our patient). In patients with stroke/transient ischemic attack (Table 1), vascular involvement of other arteries was found in four patients (aorta in 4 cases, renal arteries in 2 cases, and coronary arteries in 1 case). This observation is consistent with other studies that found periaortic fibrosis in more than half of the patients with ECD (aorta branches and other arteries being affected in only one fifth of the patients).^[22]^ Nevertheless, if cardiovascular involvement (heart failure, renovascular hypertension, and pericarditis possibly leading to tamponade) are also quite frequent in ECD (found in a third of the patients),^[22]^ stroke are surprisingly unusual in this disease.

**Table 1:**
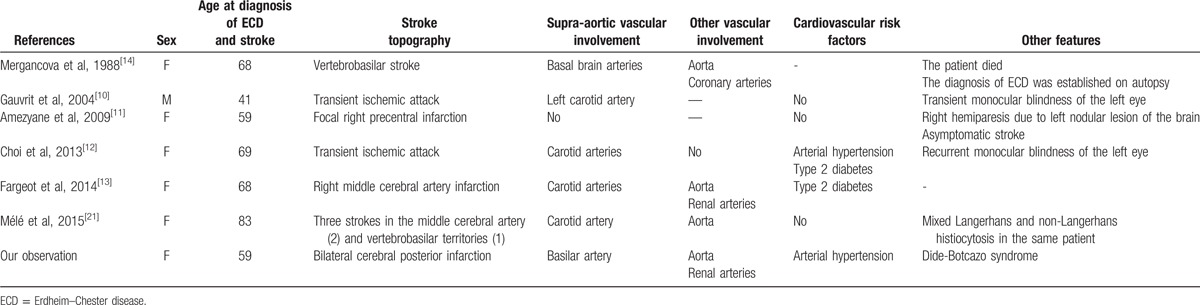
Patients with stroke and ECD.

The pathophysiological processes explaining stroke in ECD are uncertain. Few patients have the classical cardiovascular risk factors: arterial hypertension in 2 patients, and type 2 diabetes mellitus in 2 patients. Even if a coincidence is not excluded, 1 evident explanation could be the periarterial infiltration of some supra-aortic arteries, such as in our patient.^[10,12]^ It is probable that the slowness of the narrowing of the cerebral and/or cranial arteries could facilitate the adaptation of the blood circulation in the circle of Willis, except in some particular cases such as the severe stenosis of the basilar artery observed in our case. Finally, another explanation may be a decompensation of one of the potential cardiovascular involvements due to the disease (ECD can provoke renovascular hypertension or cardiopathy); our patient has renovascular hypertension but was treated by surgery 4 years earlier.

This wide clinical spectrum of ECD makes its management and treatment complicated. Many therapeutics have been tried in ECD (steroids, interferon-α, cladribine, recombinant human interleukin-1 receptor antagonist, tyrosine kinase inhibitors, bisphosphonates, and autologous hematopoietic stem cell transplantation), but interferon-α seems to be a more efficient means by improving survival in ECD patients.^[18,23]^ Recently, it has been demonstrated that vemurafenib may be also an efficient treatment in *BRAF*-mutated histiocytosis.^[24]^ In our case (partially described in another study),^[23]^ it is interesting to highlight the clinical long-term improvement (and also the dramatic recanalization of the basilar artery) after treatment (confirming that stroke was a direct consequence of the infiltration of the basilar artery). Finally, it is of the utmost importance for internists, cardiologists, and neurologists to know that stroke is a treatable and potentially reversible complication of ECD, particularly in the case of periarterial stenosis of cerebral arteries (on brain imaging) with contrast enhancement on ^18^FDG PET.
